# The possible causes for tomography suspect Keratoconus in a Chinese cohort

**DOI:** 10.1186/s12886-021-01806-9

**Published:** 2021-01-19

**Authors:** Kang Feng, Yu Zhang, Yue-guo Chen

**Affiliations:** 1 Department of Ophthalmology, Peking University Third Hospital, 49 North Garden Road, Haidian District, Beijing, 100191 People’s Republic of China; 2 Beijing Key Laboratory of Restoration of Damaged Ocular Nerve, Beijing, China

**Keywords:** Tomography suspect keratoconus, Sirius, Pentacam, Corneal horizontal diameter, Central corneal thickness

## Abstract

**Background:**

To explore the possible causes of tomography suspect keratoconus (TSK) marked by Tomography in screening keratoconus in a Chinese cohort, and the reasonable range of corneal horizontal diameter and thickness for decreasing the proportion of TSK.

**Methods:**

Nested case-control study from a single center prospective cohort. All subjects were selected from the Peking University Third Hospital Ectasia Cornea Disease Cohort Project database, which included myopic patients seeking corneal refractive surgical corrections since 2013. Demographic information, basic eye examination, and auxiliary equipment examination including refraction, IOL-master, Pentacam, Sirius, and Topolyzer were recorded. In this study, all cases were classified into two groups: TSK group and normal control (NC) group, and all of them were followed up at least 2 years. The former is consisted of those whose screening examinations of tomography are abnormal, the latter is those whose screening examinations are normal. All of them have already been followed up at least 2 years without abnormalities after excimer laser corneal refractive surgeries. *Unpaired t* tests and *Chi-square* tests were used to compare the differences of indices from the tomography between the two groups.

**Results:**

Of 183 TSK eyes (109 patients) and 160 NC eyes (83 patients), the mean age is 28.0 and 26.3 years old respectively. The corneal horizontal diameter is 11.5–11.8 mm in TSK group and 11.8–12.0 mm in NC group. The central corneal thickness is nearly 520 μm in the former and 550 μm in the latter. For Sirius, the TSK ratio of indices of SIf and SIb is 41.5 and 39.9% respectively in TSK group. For Pentacam, the TSK ratio of index IHD is 59.0% and “final D” is 72.7%.

**Conclusions:**

Corneal horizontal diameter and central corneal thickness have great influences on the results of corneal tomography in detecting the suspect keratoconus.

## Introduction

Keratoconus (KC), is one of the most important bilateral diseases of the cornea, in which the central portion of the cornea becomes thinner and bulges forward in a cone-shaped fashion resulting in myopia, irregular astigmatism, and eventually visual impairment [1]. KC is one of the most common causes of the corneal transplantation [2], and it is not rare in the world following the corneal tomography and topography were widely applicated. In Norway [3], the estimated prevalence in the general population was 192.1 per 100,000, and the estimated annual incidence was 19.8 per 100,000. In New Zealand [4], a population-based prospective cross-sectional study showed that keratoconus may affect up to 1 in 191 New Zealand adolescents. A nationwide, population-based, retrospective study of keratoconus in the entire South Korean population [5], the incidence rate in the general population was 5.56 cases per 100,000 person-years, and the incidence peaked among men in their late 20s and among women in their early 20s. In Middle East and Central Asia, Gomes [6] reported a prevalence of 2.3% in India, and Godefrooij [7] reported a prevalence of 3.18% in a population-based study of Israeli Arabs.

It’s very important to screen suspect keratoconus preoperatively, as corneal refractive surgery becomes more and more popular in the world. Previous screening studies, which were based on findings with older diagnostic modalities, had a high false negative rate. More recent studies using corneal tomography provide more sensitive estimates of prevalence/incidence [8] with the advent of Scheimpflug technology, such as Pentacam and Sirius. One of the most important indices is the final “D” value of Belin/Ambrosio Enhanced Ectasia from Pentacam [9, 10]. The Sirius combined the Placido disc and Scheimpflug technique, final class of “normal / suspect / KC” was given through a proprietary process analysis. But both of the built-in automatic analysis programs are generated mainly based on the North and South American [11] and European cases. Furthermore, the increasing sensitivity will cause tomography suspect keratoconus (TSK), which makes patients lose the opportunity for corneal refractive surgeries and invest unnecessary expenses in their reexaminations.

It is widely accepted that there are variations of tomographic parameters between the geographic and ethnic populations [12–15], and a previous publication has demonstrated the influence of corneal diameter on the individual parameters and the final “D” from Pentacam [12]. Therefore, more TSK cases are inevitable following the widespread use of this technology mentioned above in Chinese in screening suspect KC before the corneal refractive surgeries.

In this study, we attempt to discern the possible indices, which result in TSK, obtained from Pentacam and Sirius based on a Chinese cohort. Also, this study explores the reasonable range of horizontal corneal diameter and pachymetry readings, based on the screening results which can be more accurate for Chinese refractive surgery candidates.

## Methods and patients

The study conformed to the Declaration of Helsinki and was approved by the review board/ethics committee of the Peking University Third Hospital. A review of all patients was conducted and informed consents were obtained from the participants.

The cases in this study came from a cohort project – Peking University Third Hospital Ectasia Cornea Disease Cohort Project, which is a single center prospective cohort based on the outpatient of Peking University Third Hospital. It is also a referral teaching infirmary in Beijing, China. The Project was established in 2013, the data are from the patients who came to Peking University Third Hospital Eye Center seeking refractive surgeries and consented to join in the cohort study. All patients had a normal ocular examination including the slit lamp, direct and indirect ophthalmoscopy, corneal fluorescence staining, pupil size measurement, mydriatic refraction and muscle balance. All cases had a corrected distance visual acuity and Jaeger near vision, demographic information including ages, history of refractive error, ocular surgery history, and family history. Besides, auxiliary examination included IOL Master (Zeiss, Germany), Sirius (Costruzione Strumenti Oftalmici, Florence, Italy), Pentacam (Oculus, Wetzlar, Germany), Allegro Topolyzer (Alcon Inc., TX, USA), and anterior OCT (Zeiss, Germany) in necessity. All the examination results of the patients were analyzed by a professor who is also the chief-surgeon of refractive surgery and he decided whether the subject is suitable for refractive surgeries. All cases were followed up regularly in the first 6 months and the necessary examinations were applied at the visit, and the planned longest interval time of follow up is 2 years. All data files of baseline and follow-ups were collected by an assigned employer and the data was input in Microsoft Excel data frame by two assistants.

From Jan 1st, 2013 to Dec 31th, 2019, a total of 710 cases (1416 eyes) were in the cohort project database. In this study, all cases were selected from the cohort project database, the inclusion criterion and exclusion criterion were as follows:

### Inclusion criterion

Tomography suspect keratoconus (**TSK**) group, or false positive group (**FPG**), eyes marked “suspect KC” by Sirius, or marked with “yellow or red” for final “D” value in Pentacam, and those have not developed keratoconus after 2 years of follow-up following laser corneal refractive surgeries (LASEK, FS-LASIK or SMILE); Normal control (NC) group, or true negative group (TNG), eyes with all indices of Sirius are normal and all the indices Df/Db/Dp/Dt/Da/D of Pentacam are normal (marked with white), and have not developed keratoconus after 2 years of follow-up following one of the above mentioned corneal refractive surgeries. Exclusion criterion: Eyes with incomplete baseline information, or without records of follow-up.

### Keratoconus diagnosis

referred to the criterion [6], clinical diagnosis for keratoconus by one chief professor doctor based on the comprehensive judgment. In this study we conformed the criterion: One eye is diagnosed of keratoconus, the contralateral eye can be diagnosed of KC if Sirius and Pentacam indicate “KC”; One eye is diagnosed of KC, the contralateral eye can be diagnosed of VAE-N if indices of both the Sirius and Pentacam are nomal. Those eyes were excluded from any corneal refractive surgeries.

The auxiliary examination variables used in this study are as follows, all the examinations are repeated for 3–4 times for Sirius, Pentacam, and Toplyzer:

Measurement indices include HVID (horizontal visible iris diameter), Apex Curvature, MPP (mean pupil power), Anterior chamber depth, Iridocorneal angle, Corneal volume, ThkMin (minimum thickness) in Sirius; CCT (central corneal thickness) measured by A-ultrasonic and Pentacam, ThkMin in Pentacam, RMin (minimum sagittal curvature) in Pantacam; and cornea diameter in Topolyzer.

The ranked indices from Sirius, Pentacam and Topolyzer marking different colors according to the value, which were described as follows and the color predicted by machine means the possible diagnosis for subject, red means KC, yellow means suspect KC, and white means normal.

In Sirius, SIf (symmetry index of front corneal curvature) < 0.85 (white), 0.85–1.25 (yellow), > 1.25 (red); SIb (symmetry index of back corneal curvature) < 0.22 (white), 0.22–0.37 (yellow), > 0.37 (red); BCVf (Baiocchi Calossi Versaci front) < 0.80 (white), 0.80–1.20 (yellow), > 1.20 (red); BCVb (Baiocchi Calossi Versaci back) < 0.80 (white), 0.80–1.20 (yellow), > 1.20 (red); KVf (keratoconus vertex front) < 15um (white), > = 15um (red); KVb (keratoconus vertex back) < 15um (white), > = 15um (red); ThkMin < 471 (white), 471–482 (yellow), > 482 (red).

In Pentacam, the difference index comparing with normal cornea including Df (deviation of front elevation difference map), Db (deviation of back elevation difference map), Dp (deviation of average pachymetric progression index), Dt (deviation of minimum thickness), Da (deviation of Ambrosio’s Relational Thickness maximum), D (Belin/Ambrosio enhanced ectasia total derivation value), ThkMin (minimum thickness), ISV (index of surface variance), IVA (index of vertical asymmetry), KI (keratoconus index), IHA (index of height asymmetry), IHD (index of height decentration) were marked with white, yellow, or red; and CKI (center keratoconus index), RMin (minimum sagittal curvature) were marked with white or red.

In Topolyzer, ISV, IVA, KI, CKI, IHA, IHD were marked with white, yellow, or red; RMin, ABR (zernike: aberration coefficient) were marked with white or red.

### Statistical analysis

Statistical description includes the calculation of the mean, standard deviation, and 95% confidence intervals. Unpaired student *t* test was used for measurement indices and Chi square test was used for numerate variables. Statistical significance was defined as *P* < 0.05. All the eligible data were analyzed using IBM SPSS Version 26 statistic software (IBM SPSS Inc. Chicago, Illinois, USA).

## Results

In this study, total 183 TSK eyes (109 patients) and 160 NC eyes (83 patients) conform to the inclusion criterion. The mean age is 28.0 ± 6.8, and 26.3 ± 7.2 years old in TSK group and NC group respectively. There were 48 eyes in 30 males and 135 eyes in 79 females in TSK group, and 83 eyes in 43 males and 77 eyes in 40 females in NC group.

The mean spherical equivalent was − 5.86 D and − 6.28 D in TSK group and NC group (*P* = 0.04) respectively. There is no difference between them in best corrected visual acuity (BCVA) (Table 1).

**Table 1:** Difference in measurement data of parameters between True Negative Group and False Positive Group

	Mean ± SD		*P*
	TNG	FPG	
SE	−6.28 ± 1.99	−5.86 ± 1.74	0.04
SL	−5.78 ± 1.97	−5.40 ± 1.75	0.06
CL	−1.00 ± 0.74	−0.93 ± 0.80	0.37
BCVA	1.12 ± 0.10	1.12 ± 0.11	0.64
*AUS*			
CCT	551.64 ± 23.46	520.18 ± 24.39	< 0.001
*Sirius*			
HVID	12.04 ± 0.38	11.78 ± 0.37	< 0.001
AC	45.13 ± 2.33	45.94 ± 2.28	0.001
MPP	42.80 ± 1.18	43.58 ± 1.44	< 0.001
ACD	3.28 ± 0.29	3.26 ± 0.25	0.60
IA	43.98 ± 7.36	44.56 ± 4.72	0.38
CV	58.81 ± 2.50	56.60 ± 3.00	< 0.001
ThkMin	550.09 ± 23.42	520.28 ± 25.49	< 0.001
*Pentacam*			
CCT	554.38 ± 22.38	525.96 ± 25.04	< 0.001
ThkMin	549.78 ± 22.40	519.73 ± 24.84	< 0.001
RMin	7.60 ± 0.25	7.48 ± 0.27	< 0.001
*Topolyzer*			
CD	11.75 ± 0.37	11.53 ± 0.36	< 0.001

*SD* standard deviation, *TNG* True Negative Group, *FPG* False Positive Group, *KC* keratoconus, *SE* spherical equivalent, *SL* Spherical Lens degree, *CL* Cylinder Lens, *BCVA* Best corrected VA, *AUS* A-ultrasound, *CCT* central corneal thickness, *HVID* horizontal visible iris diameter, *AC* Apex Curvature, *MPP* mean pupil power, *ACD* anterior chamber depth, *IA* iridocorneal angle, *CV* corneal volume, *ThkMin* minimum thickness, *RMin* minimum sagittal curvature, *CD* cornea diameter

*After Levene test, and select corresponding two-tailed *P* value. Dependent sample t test was used

**Fig. 1:**
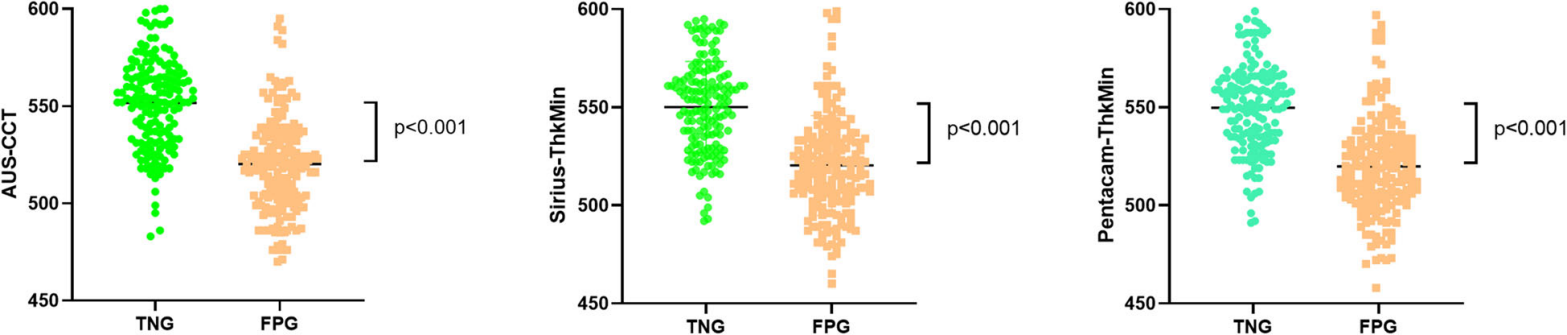
[left] Compare the horizontal visible iris diameter (HVID) measured by Sirius between true negative group (TNG) and false positive group (FPG). [right] Compare the corneal diameter (CD) measured by Topolyzer between true negative group (TNG) and false positive group (FPG). There is statistical significant difference (*p* < 0.001) for mean between the two groups in HVID and Topolyzer-CD

The HVID of Sirius are 11.8 ± 0.4 mm and 12.0 ± 0.4 mm in TSK group and NC group respectively (*P* < 0.001, Fig. 1).

**Fig. 2.**
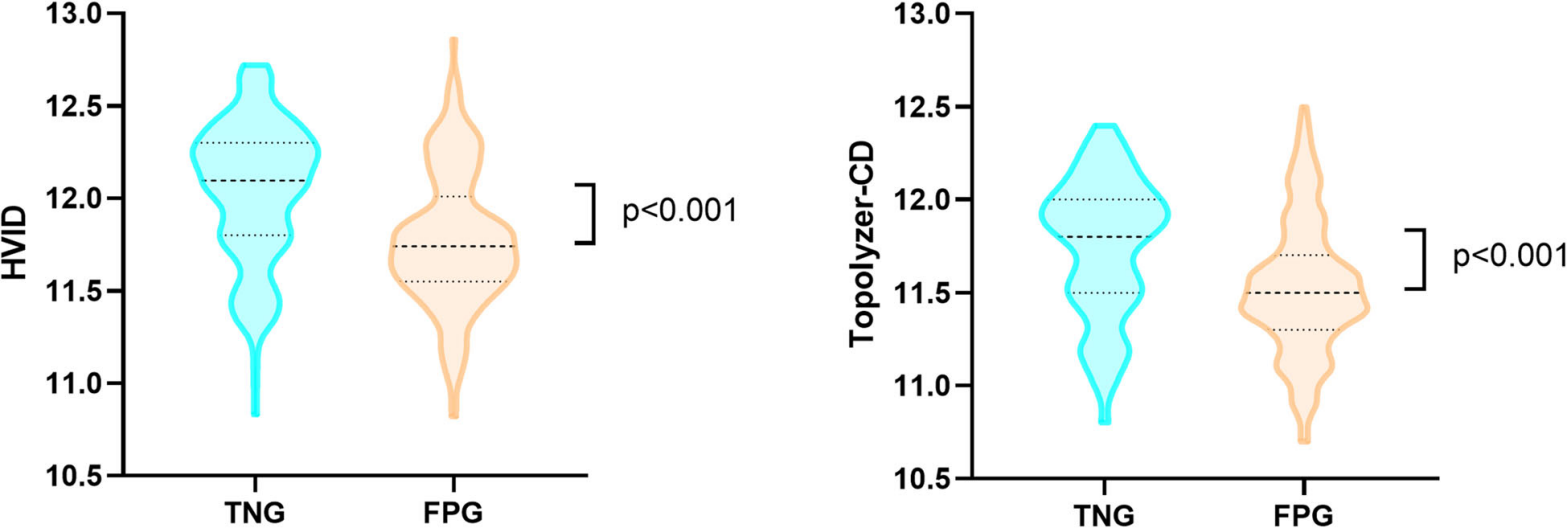
[Left] Compare the central corneal thickness measured by A-ultrasonic (AUS-CCT) between true negative group (TNG) and false positive group (FPG). There is statistical significant difference (*p* < 0.001) for mean between the two groups. [Middle] Compare the minimum thickness (ThkMin) measured by Sirius between true negative group (TNG) and false positive group (FPG). There is statistical significant difference (*p* < 0.001) for mean between the two groups. [Right] Compare the minimum thickness (ThkMin) measured by Pentacam between true negative group (TNG) and false positive group (FPG). There is statistical significant difference (p < 0.001) for mean between the two groups

The mean of Apex Curvature is 45.9 ± 2.3 D and 45.1 ± 2.3 D in TSK group and NC group respectively (*P* = 0.001). The mean of A-ultrasound for CCT is 520.18 ± 24.39 μm and 551.79 ± 23.48 μm in TSK group and NC group (*P* < 0.001, Fig. 2). The mean of central cornea thickness examined by Pentacam is 525.96 ± 25.04 μm and 554.38 ± 22.38 μm (*P* < 0.001, Fig. 2.), and the mean of thinnest thickness of the cornea is 519.73 ± 24.84 μm and 549.78 ± 22.40 μm (*P* < 0.001) in TSK group and NC group respectively.

The mean of cornea horizontal diameter measured by Topolyzer is 11.53 ± 0.36 mm and 11.75 ± 0.37 mm (*P* < 0.001) in TSK group and NC group respectively (see Table 1 and Fig. 1).

The categorical variables and statistical results for the differences of proportion between the TSK group and the NC group were listed in Table 2. (indices from Sirius), Table 3. (indices from Pentacam), and Table 4 (indices from Topolyzer).

**Table 2:** Difference in counting data of parameters of Sirius between True Negative Group and False Positive Group

		TNG		FPG		P*
		N	(%)	N	(%)	
SIf	normal	160	(100.0)	107	(58.5)	< 0.001
	suspect	0	(0.0)	52	(28.4)	
	KC	0	(0.0)	24	(13.1)	
SIb	normal	158	(98.8)	110	(60.1)	< 0.001
	suspect	2	(1.3)	65	(35.5)	
	KC	0	(0.0)	8	(4.4)	
BCVf	normal	160	(100.0)	176	(96.2)	0.016
	suspect	0	(0.0)	7	(3.8)	
	KC	0	(0.0)	0	(0.0)	
BCVb	normal	159	(99.4)	163	(89.1)	< 0.001
	suspect	1	(0.6)	16	(8.7)	
	KC	0	(0.0)	4	(2.2)	
KVf	normal	160	(100.0)	183	(100.0)	–
	abnormal	0	(0.0)	0	(0.0)	
KVb	normal	144	(90.0)	117	(63.9)	< 0.001
	abnormal	16	(10.0)	66	(36.1)	
ThkMin	< 471	0	(0.0)	2	(1.1)	0.005
	471–482	0	(0.0)	8	(4.4)	
	> 482	160	(100.0)	173	(94.5)	
Class	Normal	160	(100.0)	75	(41.0)	< 0.001
	SK	0	(0.0)	108	(59.0)	

*TNG* True Negative Group, *FPG* False Positive Group, *KC* keratoconus, *SIf* symmetry index of front corneal curvature (< 0.85 normal, 0.85–1.25 suspect, > 1.25 KC); SIb, symmetry index of back corneal curvature (< 0.22 normal, 0.22–0.37 suspect, > 0.37 KC); BCVf, Baiocchi Calossi Versaci front (< 0.80 normal, 0.80–1.20 suspect, > 1.20 KC); BCVb, Baiocchi Calossi Versaci back (< 0.80 normal, 0.80–1.20 suspect, > 1.20 KC); KVf, keratoconus vertex front (<15um normal, > = 15um abnormal); KVb, keratoconus vertex back (<15um normal, > = 15um abnormal); ThkMin, minimum thickness

* Pearson Chi square test was used, fisher exact test was used if number of one grid less than 1

**Table 3:** Difference in counting data of parameters of Pentacam between True Negative Group and False Positive Group

		TNG		FPG		
		N	(%)	N	(%)	P*
Df	normal	155	(96.9)	125	(68.3)	< 0.001
	suspect	5	(3.1)	53	(29.0)	
	KC	0	(0.0)	5	(2.7)	
Db	normal	159	(99.4)	166	(90.7)	0.001
	suspect	1	(0.6)	14	(7.7)	
	KC	0	(0.0)	3	(1.6)	
Dp	normal	154	(96.3)	129	(70.5)	< 0.001
	suspect	6	(3.8)	46	(25.1)	
	KC	0	(0.0)	8	(4.4)	
Dt	normal	160	(100.0)	167	(91.3)	0.001
	suspect	0	(0.0)	15	(8.2)	
	KC	0	(0.0)	1	(0.5)	
Da	normal	159	(99.4)	160	(87.4)	< 0.001
	suspect	1	(0.6)	23	(12.6)	
	KC	0	(0.0)	0	(0.0)	
D	normal	160	(100.0)	50	(27.3)	< 0.001
	suspect	0	(0.0)	133	(72.7)	
	KC	0	(0.0)	0	(0.0)	
ThkMin	normal	160	(100.0)	167	(91.3)	< 0.001
	suspect	0	(0.0)	15	(8.2)	
	KC	0	(0.0)	1	(0.5)	
ISV	normal	160	(100.0)	183	(100.0)	–
	suspect	0	(0.0)	0	(0.0)	
	KC	0	(0.0)	0	(0.0)	
IVA	normal	159	(99.4)	179	(97.8)	0.377
	suspect	1	(0.6)	4	(2.2)	
	KC	0	(0.0)	0	(0.0)	
KI	normal	158	(98.8)	152	(83.1)	< 0.001
	suspect	2	(1.3)	17	(9.3)	
	KC	0	(0.0)	14	(7.7)	
CKI	normal	160	(100.0)	183	(100.0)	–
	KC	0	(0.0)	0	(0.0)	
IHA	normal	159	(99.4)	178	(97.3)	0.126
	suspect	0	(0.0)	4	(2.2)	
	KC	1	(0.6)	1	(0.5)	
IHD	normal	124	(77.5)	75	(41.0)	< 0.001
	suspect	11	(6.9)	22	(12.0)	
	KC	25	(15.6)	86	(47.0)	
RMin	normal	160	(100.0)	183	(100.0)	–
	KC	0	(0.0)	0	(0.0)	
Class	Normal	157	(98.1)	147	(80.3)	< 0.001
	possible	3	(1.9)	35	(19.1)	
	KC	0	(0.0)	1	(0.5)	

*TNG* True Negative Group, *FPG* False Positive Group, *KC* keratoconus, *Df* deviation of front elevation difference map, *Db* deviation of back elevation difference map, *Dp* deviation of average pachymetric progression index, *Dt* deviation of minimum thickness, *Da* deviation of Ambrosio’s Relational Thickness maximum, *D* Belin/Ambrosio enhanced ectasia total derivation value, *ThkMin* minimum thickness, *ISV* index of surface variance, *IVA* index of vertical asymmetry, *KI* keratoconus index, *IHA* index of height asymmetry, *IHD* index of height decentration, *CKI* center keratoconus index, *RMin* minimum sagittal curvature

* Pearson Chi square test was used, fisher exact test was used if number of one grid less than1

**Table 4:** Difference in counting data of parameters of Topolyzer between true negative group and false positive group

		TNG		FPG		
		N	(%)	N	(%)	P*
ISV	normal	158	(98.8)	173	(94.5)	0.097
	suspect	1	(0.6)	7	(3.8)	
	KC	1	(0.6)	3	(1.6)	
IVA	normal	159	(99.4)	156	(85.2)	< 0.001
	suspect	1	(0.6)	15	(8.2)	
	KC	0	(0.0)	12	(6.6)	
KI	normal	156	(97.5)	135	(73.8)	< 0.001
	suspect	2	(1.3)	17	(9.3)	
	KC	2	(1.3)	31	(16.9)	
CKI	normal	160	(100.0)	183	(100.0)	–
	KC	0	(0.0)	0	(0.0)	
RMin	normal	160	(100.0)	183	(100.0)	–
	KC	0	(0.0)	0	(0.0)	
IHA	normal	155	(96.9)	160	(87.4)	0.003
	suspect	3	(1.9)	5	(2.7)	
	KC	2	(1.3)	18	(9.8)	
IHD	normal	159	(99.4)	176	(96.2)	0.076
	suspect	0	(0.0)	5	(2.7)	
	KC	1	(0.6)	2	(1.1)	
ABR	normal	123	(76.9)	136	(74.3)	0.583
	KC	37	(23.1)	47	(25.7)	
Class	KC	2	(1.3)	33	(18.0)	< 0.001
	Normal	158	(98.8)	150	(82.0)	

*TNG* True Negative Group, *FPG* False Positive Group, *KC* keratoconus, *ISV* index of surface variance, *IVA* index of vertical asymmetry, *KI* keratoconus index, *CKI* center keratoconus index, *RMin* minimum sagittal curvature, *IHA* index of height asymmetry, *IHD* index of height decentration, *ABR, zernike* aberration coefficient

*Pearson Chi square test was used, fisher exact test was used if number of one grid less than 1

In TSK group, the proportion of normal ones is 96.2% (176/183) in BCVf, and that is 89.1% (163/183) in BCVb (Table 2). There was statistical difference (Pearson chi2 = 6.76, *P* = 0.009) between them. The proportion of normal ones is 63.9% (117/183) in KVb and that is 100% in KVf, and there was statistical difference (Fisher’s exact, *p* < 0.001) between them. The proportion of normal ones is 68.3% (125/183) in Df, and that is 90.7% (166/183) in Db. There was statistical difference (Pearson chi2 = 28.19, *P* < 0.001) between them.

The laser corneal refractory surgeries employed for subjects in the study were listed in Table 5.

**Table 5:** The refractory surgery in the true negative group and false positive group

	TNG		FPG	
	N	(%)	N	(%)
LASEK	24	(15.0)	109	(59.6)
FS-LASIK	98	(61.3)	59	(32.2)
SMILE	38	(23.8)	15	(8.2)

*TNG* True Negative Group, *FPG* False Positive Group, *LASEK* Laser assisted Subepithelial Keratomileusis, *FS-LASIK* Femtosecond laser assisted Laser in-situ Keratomileusis, *SMILE* Small incision lenticule extraction

## Discussion

It is very important of screening accuracy for “suspicious keratoconus” for outpatients, especially before the corneal refractive surgeries. The current single corneal topography or tomography device, no matter which single or comprehensive indices are used, there are certain tomography suspect keratoconus and false negative results. Even if the bio-mechanical in vivo measurement indices of cornea are applied, there is no breakthrough change to the screening results. In the Peking University Third Hospital Ectasia Cornea Disease Cohort Project, we found that the corneal shape is still stable for more than 2 years after the corneal refractive surgeries in some cases which had been suggested with abnormalities by tomography and there were no signs of ectasia in them. This article is focus on the eyes with TSK suggested by corneal tomography, and to explore the possible causes of them.

In this study, the authors defined two groups, tomography suspect keratoconus (TSK) group and normal control (NC) group. The former was consisted of those whose screening examinations of Sirius or Pentacam are abnormal, and have already been followed up at least 2 years without abnormalities after one of the laser corneal refractive surgeries (Table 5). The latter was consisted of those whose screening examinations of Sirius or Pentacam are normal, and have already been followed up at least 2 years without abnormalities after refractory surgeries (Table 5).

In this study, the screening examination before refractive surgeries was Scheimpflug camera based tomographic analysis, which has been used for screening the possible KC for a few years around the world. The Pentacam and Sirius have become standard and widely accepted equipment in the evaluation of the preoperative refractive surgical candidates [16, 17]. Although the previous studies focused on the variations in tomographic parameters in 00different geographic and ethnic populations, including the influence of corneal diameter on the parameters [12], few studies on the causes of TSK in East Asian.

Most ophthalmologists used to focus on how to screen suspected keratoconus before corneal refractory surgeries to ensure the safety of post-operation. However, some of the patients diagnosed with TSK due to different reasons. After a long period of postoperative follow-up, we found that these patients could have stable corneal morphology even after myopic corneal refractive surgeries. They have certain characteristics, such as the feature of small corneal diameter. The use of a fixed size measurement range may cause the measurement of the corneal anterior and posterior surface deviations, leading to the judgement of “TSK” obtained from tomography. In addition, the corneal thickness is also an important factor in the correct judgement by tomography.

Previous publications [12, 18, 19] have reported that Chinese have been observed having smaller corneal diameters than North American (white and African-American) patients. Similar to the views of other ophthalmologists, we considered that the eye globes of Chinese are with a smaller corneal diameter, and the corneal surface may have a higher rate of change, for it has less distance between the thinnest point and periphery [12, 16]. Therefore, corneal diameter has more effects on some tomographic indices obtained from the Sirius and Pentacam comparing with Europeans and Americans.

In this study, corneal diameter was measured by Sirius and Toplyzer. The former is HVID (horizontal visible iris diameter) which was calculated, and the latter used Placido disc and directly measured the anterior corneal surface. Comparing the mean HVID in NC group with the TSK group in the database, the former is 12.04 and the latter is 11.78, the corneal diameter measured by Topolyzer is 11.75 and 11.53 respectively (Fig. 1), which indicates that the cornea with a relatively small diameter is easily diagnosed as suspicious KC by the machine.

The central corneal thickness or the thinnest point corneal thickness were measured by A-ultrasonic, Sirius, and Pentacam, and there were statistical differences between NC group and TSK group (see Table 1). The corneal thickness is considered as a predictor for KC, a study looking at OCT corneal thickness maps in normal Chinese schoolchildren found that the corneal thickness increases gradually from the center to the periphery and that central corneal thickness was associated with corneal curvature radius, but not with sex, age, axial length, or refraction [20]. Similar to our study, we found that the corneal in NC group is thicker than that in the TSK group (Fig. 2), which demonstrate that the corneal thickness is correlated with screening KC. Therefore, not only the corneal diameter but the corneal thickness is needed to be considered in screening KC.

In Sirius, the symmetry index of front/back corneal curvature (SIf/SIb) are nearly equivalent in screening KC. The SIf is an indice with statistical significance between NC group and TSK group, the cases present with suspect or KC in TSK group is 41.5%. Of 39.9% cases in TSK warned by SIb of Sirius and only 1.3% cases with warning of suspect KC by SIb in NC group (*p* < 0.001). From the results of the study, the parameters on the back of cornea are liable to present suspect or KC (yellow or red) such as KVf and KVb (keratoconus vertex front/back). Of 36.1% (66/183) eyes present “abnormal” in KVb in TSK, and there was no case present warning in KVf in the same group, there is statistical difference (Fisher’s exact, p < 0.001) between them. In addition, the proportion (96.2%) of normal ones in BCVf is higher than that (89.1%) in BCVb (*p* = 0.009). The proportion of normal ones in Df is lower (68.3%) than that (90.7%) in Db (*P* < 0.001). The results of this study suggested that the back surface indices of cornea more likely appeared tomography suspect keratoconus from Sirius, and the front surface indices of cornea more likely appeared TSK from Pentacam in Chinese. Therefore, the corneal diameter seems to have influences on the parameters of corneal front and back surface.

In this study, the ISV, CKI, and RMin of Pentacam did not present TSK results. Of 72.7% (133/183) present with yellow warning in Belin/Ambrosio enhanced ectasia total derivation value, and 59.0% (108/183) present with warnings in Class of Sirius. It has higher ratio of tomography suspect keratoconus in Pentacam than that in Sirius (Pearson chi2 = 7.59, *P* = 0.006). Though the current versions of the Pentacam are capable of measuring corneal diameter, the final “D” resulted from the analysis did not combine the corneal diameter. Brennan et al. [12] reported that corneal diameter had the highest magnitude and statistical significance of correlation with the pachymetric progression parameters and final “D” whether in Chinese or in Americans.

In the study, the difference of corneal diameter and thickness between TSK group and NC group have statistical significance. The cases with smaller and thinner cornea are liable present TSK than that with bigger and thicker cornea. Indices correlated with the corneal diameter are also different between the two groups. The Brennan et al. [12] reported that the corneal diameter affects North Americans and Chinese, and most profoundly on the pachymetric progression. The corneal diameter has a more influential effect on the Chinese population. In clinical perspective, the diameter and central cornea thickness of cornea is important in screening normal ones for corneal refractive surgeries.

This study suggests that incorporating corneal diameter and thickness as additional variables may make the BAD display of Pentacam more universally applicable. From the clinical perspective, a lot of cases seeking refractive surgeries may be excluded for the diagnosis of TSK if current screening parameters are always applied in China. As the number of this kind of studies increases, the program used for analysis can be combined with different corneal biological indicators and it will be more suitable for different races.

Combining the corneal diameter and thickness, we try to extrapolate basing on the data of our study: subjects with a corneal diameter of less than 12.04 mm (measured by Sirius) or 11.75 mm (measured by Topolyzer), and a corneal thickness of less than 550 μm were more likely to have TSK results, that is, a higher TSK rate of KC. We advise that clinical examination should be indispensable for screening the possible KC ones before refractive surgeries though the cornea tomography is important for early screening. Besides, large sample clinical studies are needed for exploring the reasonable corneal biological parameters to improve the accuracy of screening KC.

## References

[1] Gordon-Shaag A, Millodot M, Shneor E, Liu Y (2015). The genetic and environmental factors for keratoconus. Biomed Res Int.

[2] Siganos CS, Tsiklis NS, Miltsakakis DG, Georgiadis NS, Georgiadou IN, Kymionis GD, Pallikaris IG (2010). Changing indications for penetrating keratoplasty in Greece, 1982-2006: a multicenter study. Cornea.

[3] Kristianslund O, Hagem AM, Thorsrud A, Drolsum L. Prevalence and incidence of keratoconus in Norway: a nationwide register study. Acta Ophthalmol. 2020. 10.1111/aos.14668. Online ahead of print. 10.1111/aos.14668 33196151

[4] Papali'i-Curtin AT, Cox R, Ma T, Woods L, Covello A, Hall RC. Keratoconus Prevalence Among High School Students in New Zealand. Cornea. 2019;38(11):1382-9. 10.1097/ICO.0000000000002054 31335534

[5] Hwang S, Lim DH, Chung TY (2018). Prevalence and incidence of Keratoconus in South Korea: a Nationwide population-based study. Am J Ophthalmol.

[6] Gomes JA, Rapuano CJ, Belin MW, Ambrosio R, Ectatic D, Group of Panelists for the global Delphi panel of K (2015). Global consensus on Keratoconus diagnosis. Cornea.

[7] Godefrooij DA, de Wit GA, Uiterwaal CS, Imhof SM, Wisse RP (2017). Age-specific incidence and prevalence of Keratoconus: a Nationwide registration study. Am J Ophthalmol.

[8] Golan O, Piccinini AL, Hwang ES, De Oca Gonzalez IM, Krauthammer M, Khandelwal SS, Smadja D, Randleman JB (2019). Distinguishing highly asymmetric Keratoconus eyes using dual Scheimpflug/Placido analysis. Am J Ophthalmol.

[9] Belin MW, Khachikian SS, McGhee CN, Patel D (2010). New technology in corneal imaging. Int Ophthalmol Clin.

[10] Ambrosio R, Belin MW (2010). Imaging of the cornea: topography vs tomography. J Refract Surg.

[11] Gilani F, Cortese M, Ambrosio RR, Lopes B, Ramos I, Harvey EM, Belin MW (2013). Comprehensive anterior segment normal values generated by rotating Scheimpflug tomography. J Cataract Refract Surg.

[12] Boyd BM, Bai J, Borgstrom M, Belin MW (2020). Comparison of Chinese and north American tomographic parameters and the implications for refractive surgery screening. Asia Pac J Ophthalmol (Phila)..

[13] Feng MT, Kim JT, Ambrosio R, Belin MW, Grewal SP, Yan W, Shaheen MS, Jordan CA, McGhee C, Maeda N (2012). International values of central Pachymetry in Normal subjects by rotating Scheimpflug camera. Asia Pac J Ophthalmol (Phila).

[14] Feng MT, Belin MW, Ambrosio R, Grewal SP, Yan W, Shaheen MS, McGhee C, Maeda N, Neuhann TH, Burkhard Dick H (2011). Anterior chamber depth in normal subjects by rotating scheimpflug imaging. Saudi J Ophthalmol.

[15] Feng MT, Belin MW, Ambrosio R, Grewal SP, Yan W, Shaheen MS, Jordon CA, McGhee C, Maeda N, Neuhann TH (2011). International values of corneal elevation in normal subjects by rotating Scheimpflug camera. J Cataract Refract Surg.

[16] Khoramnia R, Rabsilber TM, Auffarth GU (2007). Central and peripheral pachymetry measurements according to age using the Pentacam rotating Scheimpflug camera. J Cataract Refract Surg.

[17] Emre S, Doganay S, Yologlu S (2007). Evaluation of anterior segment parameters in keratoconic eyes measured with the Pentacam system. J Cataract Refract Surg.

[18] Qin B, Tang M, Li Y, Zhang X, Chu R, Huang D (2012). Anterior segment dimensions in Asian and Caucasian eyes measured by optical coherence tomography. Ophthalmic Surg Lasers Imaging.

[19] Belin MW, Duncan JK (2016). Keratoconus: the ABCD grading system. Klin Monatsbl Augenheilkd.

[20] Ma Y, Zhu X, He X, Lu L, Zhu J, Zou H (2016). Corneal thickness profile and associations in Chinese children aged 7 to 15 years old. PLoS One.

