# Correction to: The possible causes for tomography suspect Keratoconus in a Chinese cohort

**DOI:** 10.1186/s12886-021-01832-7

**Published:** 2021-02-16

**Authors:** Kang Feng, Yu Zhang, Yue-guo Chen

**Affiliations:** 1 Department of Ophthalmology, Peking University Third Hospital, 49 North, Garden Road, Haidian District, Beijing, 100191 People’s Republic of China; 2 Beijing Key Laboratory of Restoration of Damaged Ocular Nerve, Beijing, China

**Correction to: BMC Ophthalmol 21, 47 (2021)**

**https://doi.org/10.1186/s12886-021-01806-9**

Following publication of the original article [1], we were notified that the figures and legends were reversed.

Furthermore, two extra characters (“00”) were mistakenly added in the second paragraph of the Discussion section.

The original article has been corrected.
